# Cytotoxicity Modulated by Cyanotoxins in Neuroblastoma SH-SY5Y Cells

**DOI:** 10.33696/pathology.6.058

**Published:** 2025

**Authors:** Suryakant Niture, Sashi Gadi, Somnath Mukhopadhyay, Deepak Kumar, Qing Cheng

**Affiliations:** 1 The Julius L. Chambers Biomedical/Biotechnology Research Institute (JLC-BBRI), North Carolina Central University (NCCU), Durham, North Carolina 27707, USA; 2 Department of Radiation Oncology, Stephenson Cancer Center, Oklahoma University, Oklahoma City, OK 73104 USA

**Keywords:** Cyanotoxins, Microcystin-LR, Nodularin, Cylindrospermopsin, β-N-methylamino-l-alanine, Neuroblastoma, Neurotoxicity

## Abstract

Aquatic prokaryotic cyanobacteria (algal blooms) produce cyanotoxins (CTs), significant pollutants in aquatic ecosystems. Direct exposure to high-concentration CTs through inhalation, skin contact, or ingestion of contaminated water can lead to hepatotoxicity and neurotoxicity. However, the effect of exposure to CTs at low concentrations remains unclear. Given that CTs can cross the blood–brain barrier via organic anion transporting polypeptides (OATPs), we investigated the effect of acute exposure to low concentrations (10 nM and 50 nM) of CTs, namely microcystin-LR (MC-LR), nodularin (NOD), cylindrospermopsin (CYN), and known neurotoxin β-N-methylamino-l-alanine (BMAA) in neuroblastoma SH-SY5Y cells. Using MTT assays, we found that all tested CTs increased cell survival at low concentrations. MC-LR, NOD, and CYN regulated the expression of metabolic *CYP1A1*, *CYP1A2*, *CYP2D6*, *CYP2E1*, *CYP3A4*, and *VEGFA* expression differentially, whereas BMAA downregulates metabolic gene expression. CT exposure downregulates mitochondrial oxygen consumption rate in neuroblastoma SH-SY5Y cells, increases *IL-6*, *SOD1*, and *TNFα* expression, and enhances cell apoptosis. CTs also downregulated unfolded protein response-associated gene expression and increased Tau phosphorylation. Collectively, these findings suggest that acute exposure to low concentrations of CTs modulates neuroblastoma cell metabolism, Inflammatory signaling, and AD-related markers, highlighting a potential link between environmental toxin exposure and neurotoxicity.

## Introduction

In recent years, global warming has become a challenging aspect contributing to rising temperatures in water reservoirs, Creating favorable conditions for aquatic algal blooms, particularly blue-green algae/cyanobacteria growth. Cyanobacteria produce cyanotoxins (CTs), which can be categorized into cyclic peptides, alkaloids, lipopeptides, non-protein amino acids, and lipoglycans [1]. CT concentrations in global water samples range from undetectable levels to 7,000 μg/L, posing a significant threat to aquatic ecosystems, irrigation systems, and drinking water supplies, and are now considered a significant class of environmental pollutants [2,3]. Among the various CTs, Microcystin-LR (MC-LR) is the most abundantly present in the environment worldwide [4], and it is one of the most potent toxins released from cyanobacteria, and can cause different pathological conditions in humans [5,6].

Human exposure to CTs can occur through skin contact, inhalation, or ingestion of contaminated water, as well as through consumption of aquatic organisms, such as fish or shellfish, that bioaccumulate CTs through the food chains [7–9]. Such exposure can cause liver and brain cytotoxicity, cardiovascular risk, as well as respiratory, dermatologic, gastrointestinal, and neurologic signs and symptoms [10–13]. CTs hepatotoxicity is well documented, such as morphological changes in hepatocytes, increased cytotoxicity, and modulation of lipogenic and fibrotic signaling in liver cells [14–16].

Efforts to control CT contamination in aquatic environments have included bacterial biodegradation [17], photolysis and advanced CTs oxidation process [18], sedimentation and absorption [19], and the use of ultra and nano-filtration systems [17]. Detoxification of CTs typically occurs through glutathione conjugation, facilitated by enzymes such as glutathione S-transferase (GST) and glutathione reductase (GR), which help reduce CTs accumulation in shrimp [20–22]. A recent study suggests that enzyme microcystinase enhances MCs biodegradation [the highest MC-LR degradation rate – (1.0 μ g/mL/min)] and may be useful promising strategy to eliminate MCs from the aquatic system [23].

In addition to hepatotoxicity, several CTs can cross the blood-brain barrier (BBB), either by disrupting its integrity or via active transport mechanisms involving organic anion transporting polypeptides (OATPs) [24,25], leading to detrimental changes in brain functions and behavioral alterations [11,26,27]. When exposed to high concentrations, CT increased oxidative stress, neuro-inflammation, neurotoxicity, protein misfolding in neuronal cells, as well as altered neurotransmission, calcium, and protein homeostasis in animal brains [28–30]. Some cyanotoxins, such as *β*-*N*-methylamino-l-alanine (BMAA), a potent neurotoxin, have been linked to neurological diseases such as ALS (amyotrophic lateral sclerosis), PD (Parkinson’s disease), and AD (Alzheimer’s disease) [28,31,32]. MC-LR-treated hippocampi showed alterations in protein expression involved in the cytoskeleton, neurodegenerative disease, oxidative stress, apoptosis, and energy metabolism [33]. MC-LR exposure resulted in tau hyperphosphorylation and neuronal degeneration, potentially contributing to AD [33]. Despite these findings, the effects of low or physiologically relevant concentrations of CTs on neuronal cytotoxicity and their potential role in the development of neurodegenerative diseases such as AD remain largely unknown.

We recently found that even at a low concentration of CT exposure can induce altered lipogenic signaling, liver cell steatosis, and cytotoxicity in liver cells [14]. Moreover, CTs not only target the liver but also contribute to brain toxicity through interaction with the gut-liver-brain axis [11,27]. In this study, we assessed the impact of low concentrations of CTs such as MC-LR, NOD, CYN, and BMAA (10 and 50 nM) on neurotoxicity in human neuroblastoma SH-SY5Y cells. We analyzed neuroblastoma cell cytotoxicity and determined changes in cell metabolic activities, inflammatory and unfolded protein response (UPR) signaling, and AD markers Tau and β-amyloid expression after acute exposure to CTs.

## Materials and Methods

### Preparation of cyanotoxins (CTs)

CTs were purchased from Enzo Life Science (Farmingdale, NY, USA). Microcystin-LR (Cat # ALX-350–012-C100) was dissolved in DMSO, Nodularin (NOD) (Cat # ALX-350–061-C100) dissolved in 50% methanol diluted with H_2_O, and Cylindrospermopsin (CYN) (Cat # ALX-350–149-C100) in methanol (100%). Neuro cyanotoxin BMAA (Cat# 2538) was obtained from Tocris Bioscience and dissolved in H_2_O. All stocks were diluted at 10 μM concentrations, and freshly prepared CTs were used for experiments. Cells were exposed to the indicated CTs, or vehicle (v/v), and the vehicle concentration was kept below 0.01%.

### Cell culture

Neuroblastoma SH-SY5Y cells were obtained from ATCC (Cat # CRL-2266) and grown in DMEM (Dulbecco’s Minimum Essential Medium) supplied with 10% fetal bovine serum (FBS), 1% penicillin/streptomycin (Pen/Strep) at 37°C in an incubator provided with 5% CO_2_. After 24–48h after plating, replace serum-containing medium with neurobasal medium (containing B27 supplement and GlutaMAX) and treat with all-trans-retinoic acid (ATRA; 5 μM) for 5 days to promote differentiation and neuronal phenotype [34]. SH-SY5Y cell line is a thrice-cloned subline of the neuroblastoma derived from a metastatic bone tumor and suitable for toxicology research. Similarly, N18TG2 cells were obtained from Sigma-Aldrich (St. Louis, MO, Cat # 08062523) and grown in DMEM /Ham’s F-12 50/50 mix supplied with 10% FBS, 1% penicillin/streptomycin (Pen/Strep), and differentiated with exposure to all-trans-retinoic acid (ATRA; 5 μM). N18TG2 (mouse neuroblastoma) cells are derived from the N18 clonal line produced from the mouse neuroblastoma C1300 (strain A/Jax), known for expressing neuronal properties such as the ability to synthesize and respond to neurotransmitters. N18TG2 cells are used to study neuronal function, neuronal differentiation, and metabolic processes. Cells were incubated at 37°C in an incubator with 5% CO_2_. Fully differentiated and more than 70% confluence cells were used for experiments.

### MTT cell survival assay

Fully differentiated neuroblastoma SH-SY5Y cells (5,000 cells/well) and N18TG2 cells were incubated in 96-well plates (Falcon, Corning, NY) for 20 h. Cells were exposed to 10 nM and 50 nM concentrations of cyanotoxins (CTs) for 72 h, and cell survival colorimetric MTT (3-(4,5-dimethylthiazol-2-yl)-2,5-diphenyl tetrazolium bromide; MP Biomedicals, Solon, OH) assay was performed as described previously [14]. Vehicle and CT-treated cells were exposed to MTT reagent 5 μl/ well (stock solution: 5 mg/mL in PBS) for 1 hour at 37°C. Cells were washed with PBS, and the formazan crystals were dissolved in DMSO (Fisher Chemical, 99.9%). Color intensity was measured at 570 nm using a FLU Ostar^®^ Omega microplate reader (BMG Lab Tech, Cary, NC).

### Western blotting

Fully differentiated neuroblastoma SH-SY5Y cells were incubated in 6 well plates (1×10^5^ cells/ well) and exposed to 10 nM concentration of CTs for 72h. Cell lysates were prepared; protein concentrations were determined using Bio-Rad protein assay dye (Cat # 5000006) as described previously [14]. For Western Blotting (WB), 60 μg of protein samples were loaded and separated on NuPAGE 4%–12% Bis-Tris-SDS gel (Invitrogen), transferred to a polyvinylidene difluoride (PVDF) membrane (Thermo Scientific, Rockford, IL), and the membranes were incubated with indicated primary antibodies overnight at 4°C. Anti-SOD1 (Cat # 2770S), anti-CAT (Cat # 12980T), anti-Cleaved PARP (Cat # 9541S), anti-β-actin (Cat # 4970S) and anti-β-tubulin (Cat # 2128S) antibodies were obtained from Cell Signaling Technology (Danvers, MA, USA). We purchased anti-β-amyloid antibodies (Cat# 44–344 & Cat # 36–6900), anti-Tau-pSer404 (Cat# 44–758G), anti-Tau-pS202-pThr205 (Cat# MN1020) from Invitrogen (Rockford, IL, USA), and anti-BDNF (Cat# AB1534SP) from Millipore. The membranes were washed with TBST and further incubated in the appropriate secondary antibody (1: 10000 dilution) (Jackson Immuno Research, PA) for 1h at room temperature. The western bots were developed using ECL chemiluminescence detection reagents (Signagen Laboratories, Rockville, MD) and the Azure C-500 Bio-system. Western blotting was repeated three times, and band intensities were quantified (https://imagej.nih.gov/ij/).

### RT/qPCR

Fully differentiated neuroblastoma SH-SY5Y cells were incubated in 6-well plates (1×10^5^ cells/ well) and exposed to 10 nM concentrations of the indicated CTs for 48h. Cells were washed with cold PBS, and total RNA was isolated using TRIZOL reagent (Invitrogen, Carlsbad, CA). Using a High-Capacity cDNA Reverse Transcription kit (Applied Biosystems, Carlsbad, CA), cDNA was prepared [14]. The cDNA was mixed with Power SYBR Green master mix (Applied Biosystems) with specific forward and reverse human primers of the inflammatory signaling genes, UPR genes, and drug/xenobiotic metabolic genes (Supplementary Table 1). All primers were obtained from Integrated DNA Technology (IDT, Coralville, IA). A human *GAPDH* set of primers was used to express the *GAPDH* gene as an internal control in the qPCR reaction. QuantStudio-3 PCR Instrumental System (Applied Biosystems) was used to run the PCR reactions. The relative gene expression and quantitation were performed according to the manufacturer’s protocols.

### Seahorse bioanalyzer

The Cell Mito-Stress Assay was used to evaluate the effect of cyanotoxins (CTs) on the mitochondrial oxygen consumption rate (OCR). Fully differentiated neuroblastoma SH-SY5Y cells were pretreated with NOD and BMAA (10 n M) for 72 h, then trypsin zed and reseeded (at 3 × 10^4^ cells/well) into XFp plates with XF media and supplements and incubated at 37°C for 18 h following the manufacturer’s instructions for the XF Cell Mito-Stress Assay. Before the assay, cells were also exposed at 37°C without CO_2_ followed by Oligomycin, FCCP and Rotenone, and Antimycin treatments, and we analyzed OCR for 90 min using Wave software (Seahorse/Agilent). Oligomycin inhibits ATP synthase (Complex V), allowing for the measurement of oxygen consumption not linked to ATP production (i.e., proton leak), and helps assess the portion of basal respiration dedicated to ATP synthesis. FCCP (Carbonyl cyanide-p-trifluoromethoxy phenylhydrazone) is a mitochondrial uncoupler that dissipates the proton gradient across the inner mitochondrial membrane. It forces the electron transport chain (ETC) to function at its maximum capacity, thus enabling measurement of maximal respiration. Rotenone and Antimycin A inhibit Complex I and Complex III, respectively, shutting down mitochondrial respiration. The remaining oxygen consumption reflects nonmitochondrial respiration.

### Statistical analysis

Results are from independent experiments (n=3) and presented as means SEM. Differences between groups were analyzed using a two-tailed Student’s *t*-test, and a p-value of <0.05 was considered statistically significant. GraphPad Prism 9 software (GraphPad Software Inc., La Jolla, CA) was used for analysis and graph plotting.

## Results

### Acute exposure CTs enhance cell survival and modulate the expression of metabolic genes in SH-SY5Y cells

**Figure 1.**
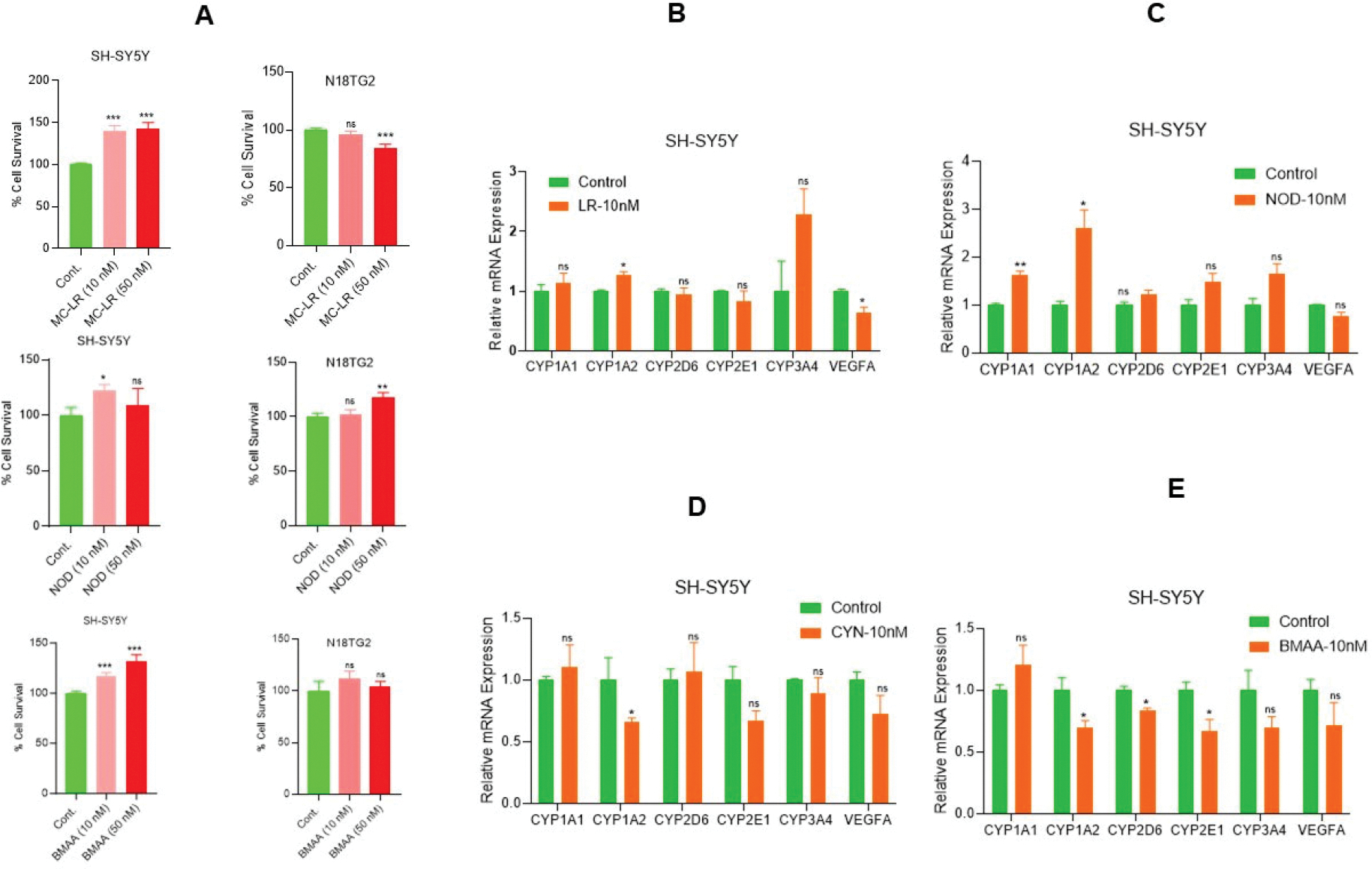
Low concentrations of CT exposure regulate cell metabolic activities in neuroblastoma. (**A**) Fully differentiated neuroblastoma SH-SY5Y cells and N18TG2 cells were cultured in 96-well plates (5000 cells/well; n=12), and after 16 h, cells were exposed to 10 nM and 50 nM concentrations of MC-LR, NOD, and BMAA for 72 h. Cell survival MTT assay was performed, and relative cell survival was analyzed. *P<0.05, **P<0.01, ***P<0.001 compared to vehicle cells. (**B-E**) SH-SY5Y cells were exposed to MC-LR, NOD, CYN, and BMAA at 10 nM concentration for 48 h, and expression of cell metabolic *CYP1A1, CYP1A2, CYP2D6, CYP2E1, CYP3A4*, and *VEGFA* gene expression was analyzed by RT/qPCR as described in the materials and methods section. *P<0.05 compared with vehicle-exposed cells. ns: Not Significant.

Cyanotoxins (CTs) are secondary metabolites from cyanobacteria that impact multiple human organs, including the liver and brain, and promote carcinogenesis [26,35]. Since normal human neuronal cells are not easily available, in this study, we used human neuroblastoma SH-SY5Y cells and mouse neuroblastoma N18TG2 cells to assess the effects of CTs on cell survival. The MTT assay demonstrated that acute exposure (72 h) to MC-LR, NOD, and known cyanotoxin/neurotoxin BMAA, even at 10 nM concentration, increased cell survival in SH-SY5Y cells (Figure 1A, left panels). Specifically, MC-LR increased by 39.97%, and NOD by 22% cell survival at 10 nM concentration compared with vehicle-treated controls (Figure 1A, left panel). BMAA exposure resulted in a 16% and 31% increase in cell survival at 10 nM and 50 nM, respectively, compared with vehicle-treated cells (Figure 1A, left panel). In N18TG2 cells, NOD at 50 nM increased cell survival by ~17% (Figure 1A, right panel), while MC-LR decreased cell survival by ~16%. No significant effects were observed in N18TG2 cells exposed to BMAA. These findings suggest that CTs modulate cell survival and proliferation in neuroblastoma cells.

To investigate the metabolic activities modulated by CTs, we analyzed the expression of metabolic genes, including *CYP1A1*, *CYP1A2*, *CYP2D6*, *CYP2E1*, *CYP3A4*, and *VEGFA* in SH-SY5Y cells after CT exposure. RT-qPCR data revealed that MC-LR increased the expression of *CYP1A1* (1.12-fold-ns), *CYP3A4* (2.28-fold-ns), *CYP1A2* (1.26-fold, significantly), while significantly decreasing *VEGFA* expression was observed compared to vehicle-exposed cells (Figure 1B). Similarly, NOD exposure upregulates *CYP1A1* (1.64-fold, significantly), *CYP1A2* (2.62-fold, significantly), *CYP2E1* (1.49-fold-ns), and *CYP3A4* (1.66-fold-ns) compared with vehicle-treated cells (Figure 1C). In contrast, CYN exposure led to a reduction in *CYP1A2* (0.66-fold, significantly), *CYP2E1* (0.67-fold-ns), *CYP3A4* (0.89-fold-ns), and *VEGFA* (0.72-fold-ns) expression in SH-SY5Y cells (Figure 1D). Similarly, BMAA exposure decreased significantly the expression of *CYP1A2*, *CYP2D6*, and *CYP2E1* (~0.67-fold), and *VEGFA* (0.71-fold-ns) compared with vehicle-treated SH-SY5Y cells (Figure 1E). Together these findings suggest that even at a concentration of 10 nM, CTs differentially regulate cell metabolism and survival in neuroblastoma cells.

### CTs altered the expression of IL-6, SOD1, and TNFα mRNAs

**Figure 2.**
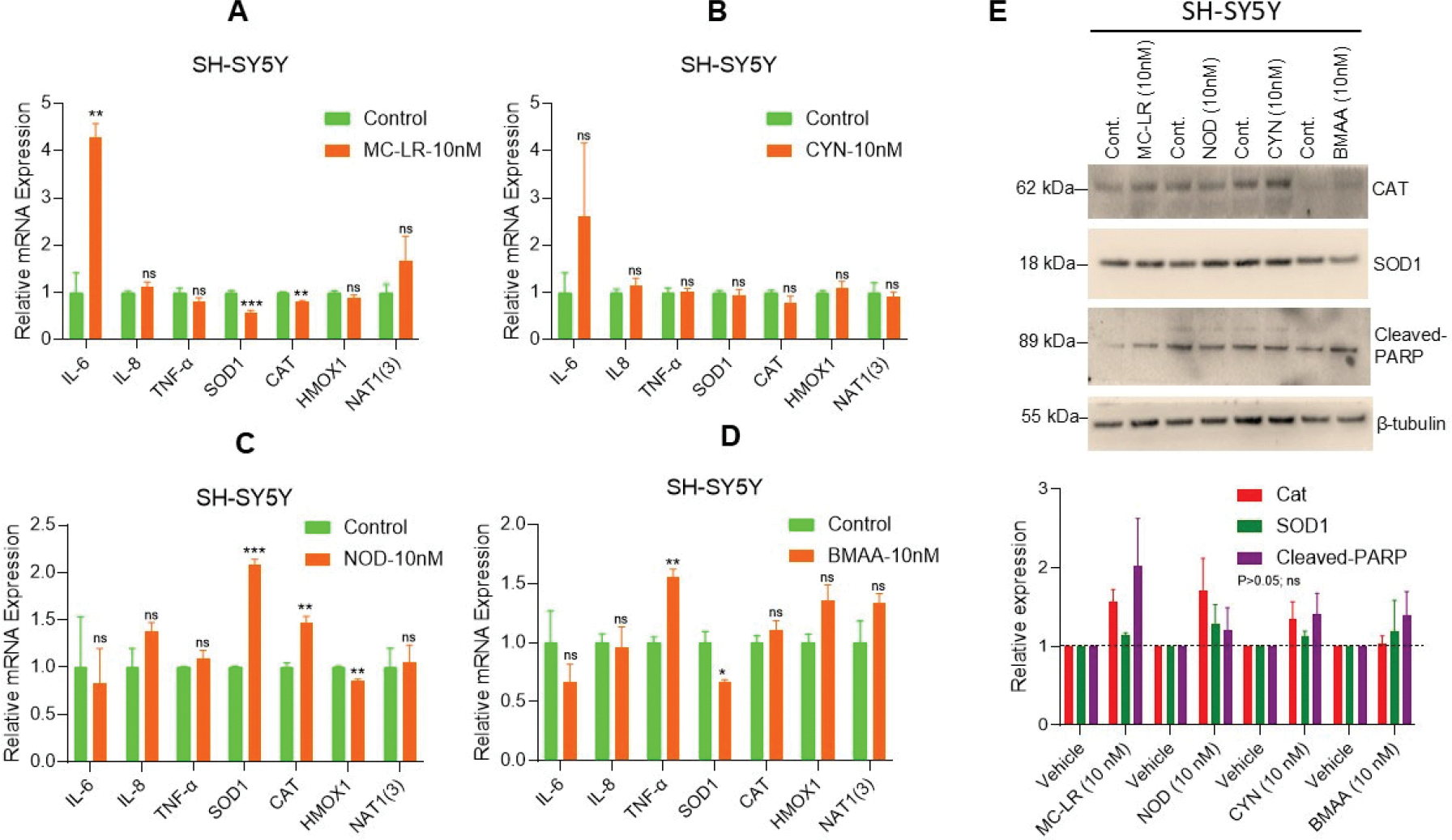
CT exposure regulates the expression of inflammatory gene mRNAs in SH-SY5Y cells. (**A–D**) Neuroblastoma SH-SY5Ycells were exposed to MC-LR, NOD, CYN, and BMAA at 10 nM concentration for 48h, and expression of *IL6, IL8, TNFα, SOD1, CAT*, *HMOX1*, and *NAT1(3)* mRNA expression was analyzed by RT/qPCR as described in the materials and methods section. *P<0.05, **P<0.01, and ***P<0.001 compared with vehicle exposed cells. (**E**) SH-SY5Y cells were exposed to MC-LR, NOD, CYN, and BMAA at 10 nM concentration for 72 h, and sixty micrograms of protein lysates were immunoblotted with anti-CAT, anti-SOD1, anti-Cleaved-PARP, and β-tubulin antibodies (upper panel). Immunoblots were repeated thrice, and band intensities were quantified (lower panel) and plotted (https://imagej.nih.gov/ij/; Version 1.53t, accessed on 25 June 2024). ns: Not Significant.

To assess CT-induced inflammation responses in neuroblastoma cells, we analyzed the expression of pro-inflammatory cytokines *IL-6*, *IL-8*, and *TNF-α* and other inflammatory proteins such as *SOD1*, *CAT*, *HMOX1*, and *NAT1(3).* Our RT-qPCR data indicates that exposure to MC-LR significantly increased *IL-6* expression (4.2-fold) while decreased the expression of *SOD1* and *CAT* compared to vehicle-treated cells (Figure 2A). MC-LR exposure does not affect the expression of *IL-8*, *TNF-α*, *HMOX1*, and *NAT1(3)* significantly (Figure 2A). Similarly, CYN exposure increased *IL-6* (2.59-fold) but not significantly compared to vehicle treated cells and no significant changes in expression of *IL-8*, *TNF-α*, *SOD1*, *CAT*, *HMOX1*, and *NAT1(3)* genes were observed (Figure 2B). NOD treatment significantly increased *SOD1* and *CAT* expression (2-fold and 1.46-fold, respectively) and significantly decreased *HMOX1* expression (0.85-fold) compared to vehicle exposed cells. No significant change in expression of *IL-6*, *IL-8*, *TNF-α*, *HMOX1*, and *NAT1(3)* mRNA was observed when the cells were exposed to NOD (10 nM) (Figure 2C). Whereas BMAA exposure significantly increased *TNF-α* mRNA level (1.55-fold) and decreased *SOD1* mRNA level (0.66-fold) compared to vehicle exposure SH-SY5Y cells and no significant change in expression of *IL-6*, *IL-8*, *CAT*, *HM*OX1, and *NAT1(3)* mRNAs were observed when the cells were exposed to BMAA (10 nM) (Figure 2D). These results suggest that CTs regulate inflammatory signaling in neuroblastoma cells.

Additionally, we analyzed the expression of CAT and SOD1 proteins following CT exposure. Immunoblotting data indicates that MC-LR, CYN, and BMAA increased but not significantly CAT (p>0.05) expression, while MC-LR, NOD, and CYN enhanced SOD1 expression but not significantly (p>0.05) (Figure 2E, upper and lower panels). Notably, both MC-LR and BMAA increased cleaved PARP expression (not significantly; p>0.05), an apoptosis marker, in neuroblastoma cells (Figure 2E, upper and lower panels). Collectively, our data suggests that acute exposure to CTs (10 n M) alters the inflammatory gene expression in neuroblastoma SH-SY5Y cells.

### The effect of CTs on mitochondrial function

**Figure 3.**
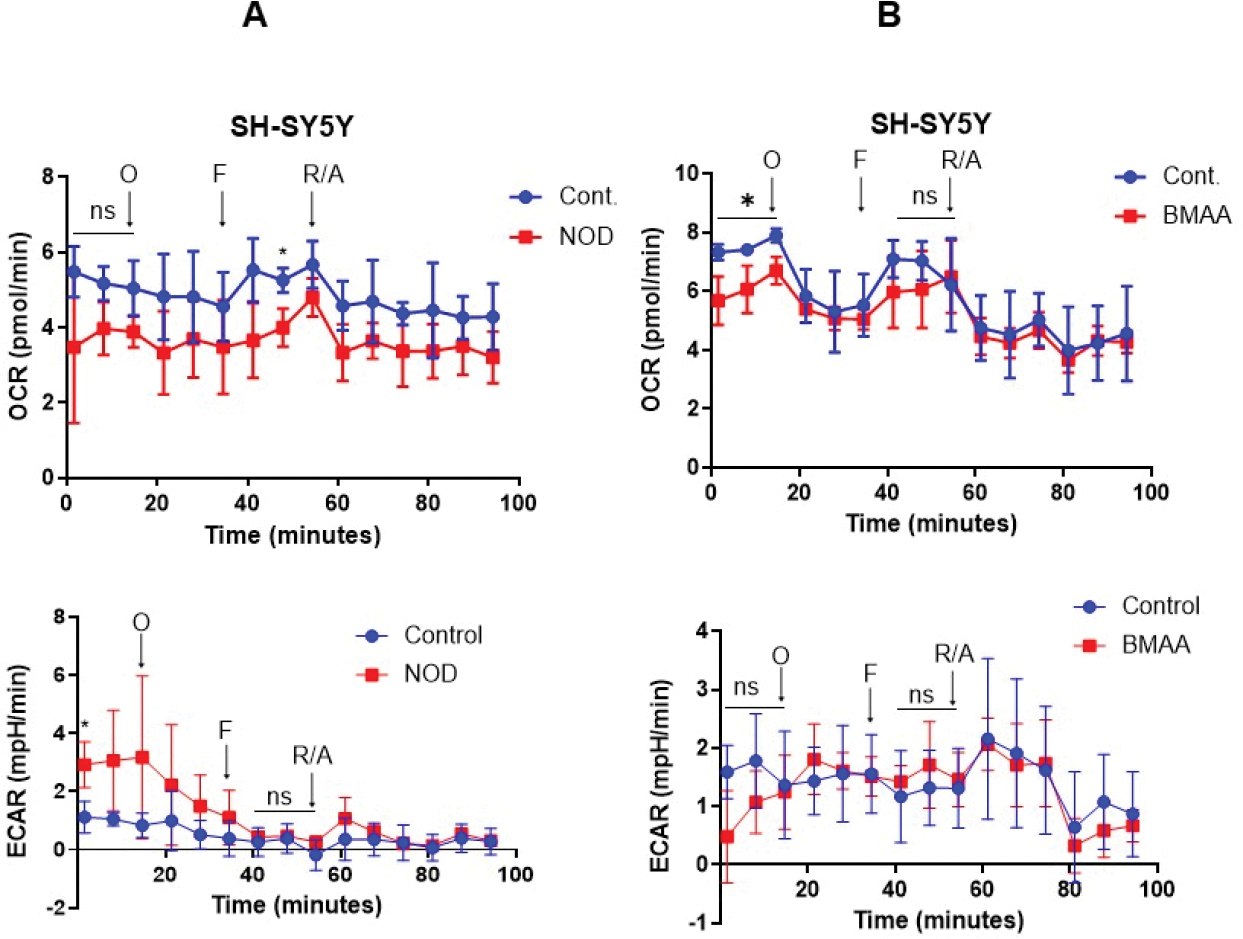
Effect of CTs on mitochondrial oxygen consumption rate and cell energy phenotype. (**A&B**) SH-SY5Y cells were exposed to CTs (10 nM) for 72 h as indicated, and after exposure cells (3 × 10^4^ cells/well) were subjected to Seahorse Bio-analyzer, and relative OCR and EACR were analyzed as described in the materials and methods section (left and right panels). *P<0.05 compared with vehicle-exposed cells. O: Oligomycin; F: FCCP; R/A: Rotenone and Antimycin A; ns: Not Significant.

To test the role of CTs on mitochondrial function, we analyzed the impact of mitochondrial oxygen consumption rate (OCR) after 72 h CTs [NOD, and BMAA (10 n M separately)] exposure using the Seahorse Cell Mito-Stress Assay. After 72 h of CTs exposure cells were further exposed to oligomycin, FCCP, and rotenone/antimycin, which are commonly used to examine mitochondrial stress/respiration/function. Oligomycin inhibits ATP synthase and allows us to measure basal respiration and the extent of energy-dependent oxygen consumption (independent of ATP), whereas FCCP acts as an uncoupler of the electron transfer chain (ETC) and increases mitochondrial membrane potential, allowing ETC to operate at its maximum capacity. Inhibition of Complex I (ETC) by rotenone and Complex III (ETC) by antimycin A allows for measuring nonmitochondrial respiration. We used the Agilent Seahorse XF bioanalyzer and Mito-stress tests for assessing mitochondrial function. Our data indicate that exposure to NOD (10 nM) significantly downregulates OCR in SH-SY5Y cells when cells are treated with FCCP (Figure 3A, upper panels), whereas BMAA (10 nM) exposure shows significantly lower OCR before exposure of cells with oligomycin and FCCP. No significant change in extracellular acidification rate (ECAR) was observed when cells were exposed to NOD and BMAA (Figures 3A and 3B, lower panels), suggesting that CTs downregulated OCR and mitochondrial respiration in neuroblastoma SH-SY5Y cells.

### CTs downregulate the unfolded protein response (UPR) gene signature

**Figure 4.**
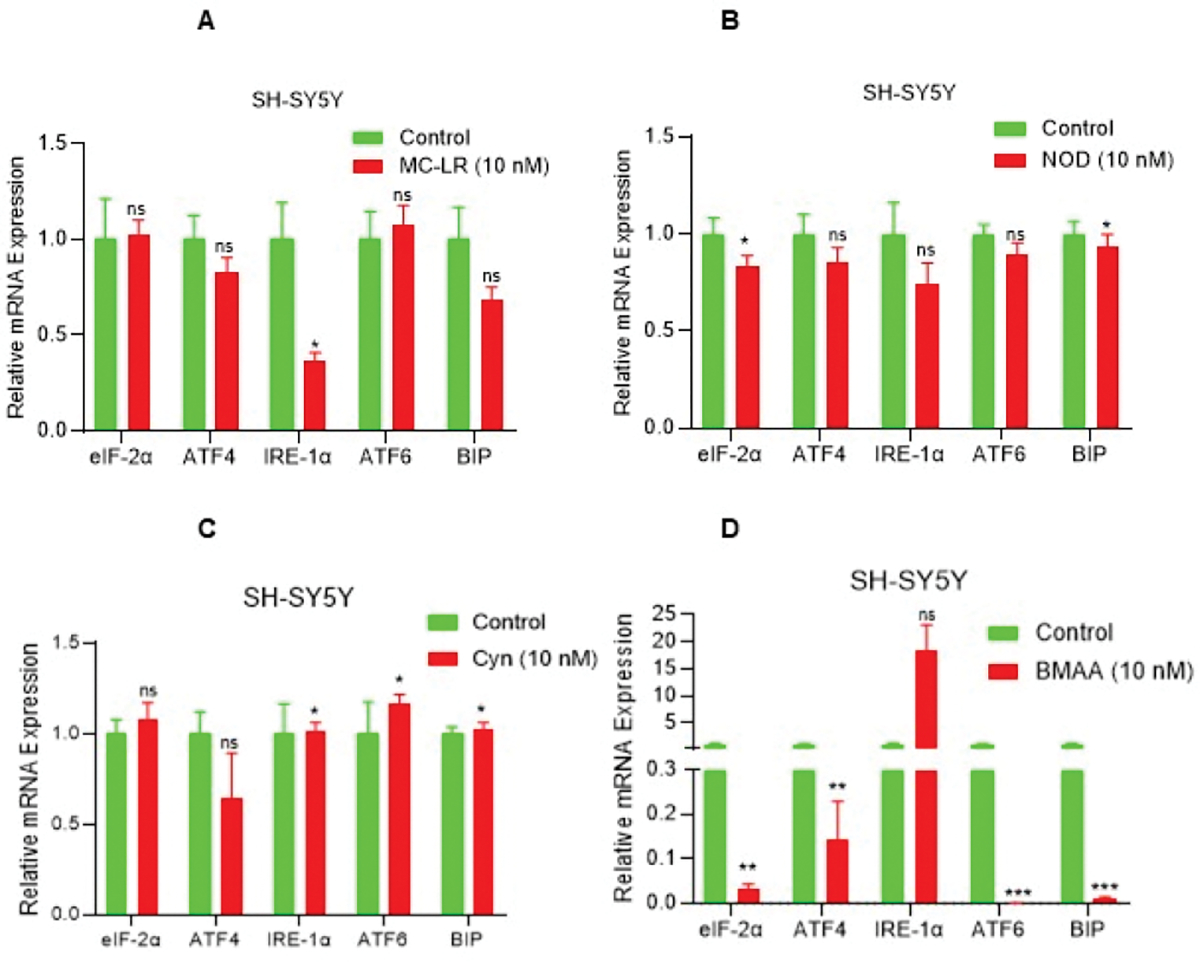
CT exposure downregulates UPR genes in SH-SY5Y cells. (**A–D**) SH-SY5Y cells were exposed to MC-LR, NOD, CYN, and BMAA at 10 nM concentration for 48 h, and expression of UPR genes such as *eIF-2α, ATF4, ATF6, IRE-1α*, and *BIP1* expression were analyzed by RT/qPCR. *P<0.05, **P<0.01 and *** *P*<0.001 compared with vehicle exposed cells. ns: Not Significant.

The unfolded protein response (UPR) pathway plays a protective role in cellular responses activated by endoplasmic reticulum (ER) stress, and also plays an important role in neurodegenerative diseases [36]. Given that CTs modulate inflammatory signaling and induce cellular stress/toxicity in neuroblastoma cells, we analyzed the expression of *UPR* gene biomarkers following exposure to CTs (10 nM). Interestingly, MC-LR exposure downregulates *IRE-1α* gene expression significantly and decreases expression of *ATF4* and *BIP* gene expression (p>0.05; ns), similarly, NOD also downregulates *eIF-2α* and *BIP* (significantly) and decreases *ATF4*, *IRE-1α*, and *ATF6* gene expression (p>0.05; ns) compared with vehicle treated cells (Figures 4A and 4B). CYN reduces *ATF4* expression (ns) and significantly increases (10 to 15%) of *IRE-1α*, *ATF6*, and *BIP* gene expression, whereas BMAA inhibits *eIF-2α*, *ATF4*, *ATF6*, and *BIP* expression (p<0.05) and increases *IRE-1α* expression (ns) (Figures 4C and 4D), overall, the data suggesting that CTs downregulate *UPR* gene expression and thus enhance cytotoxicity in SH-SY5Y cells.

### The effect of CTs on the expression of AD pathologic markers

**Figure 5.**
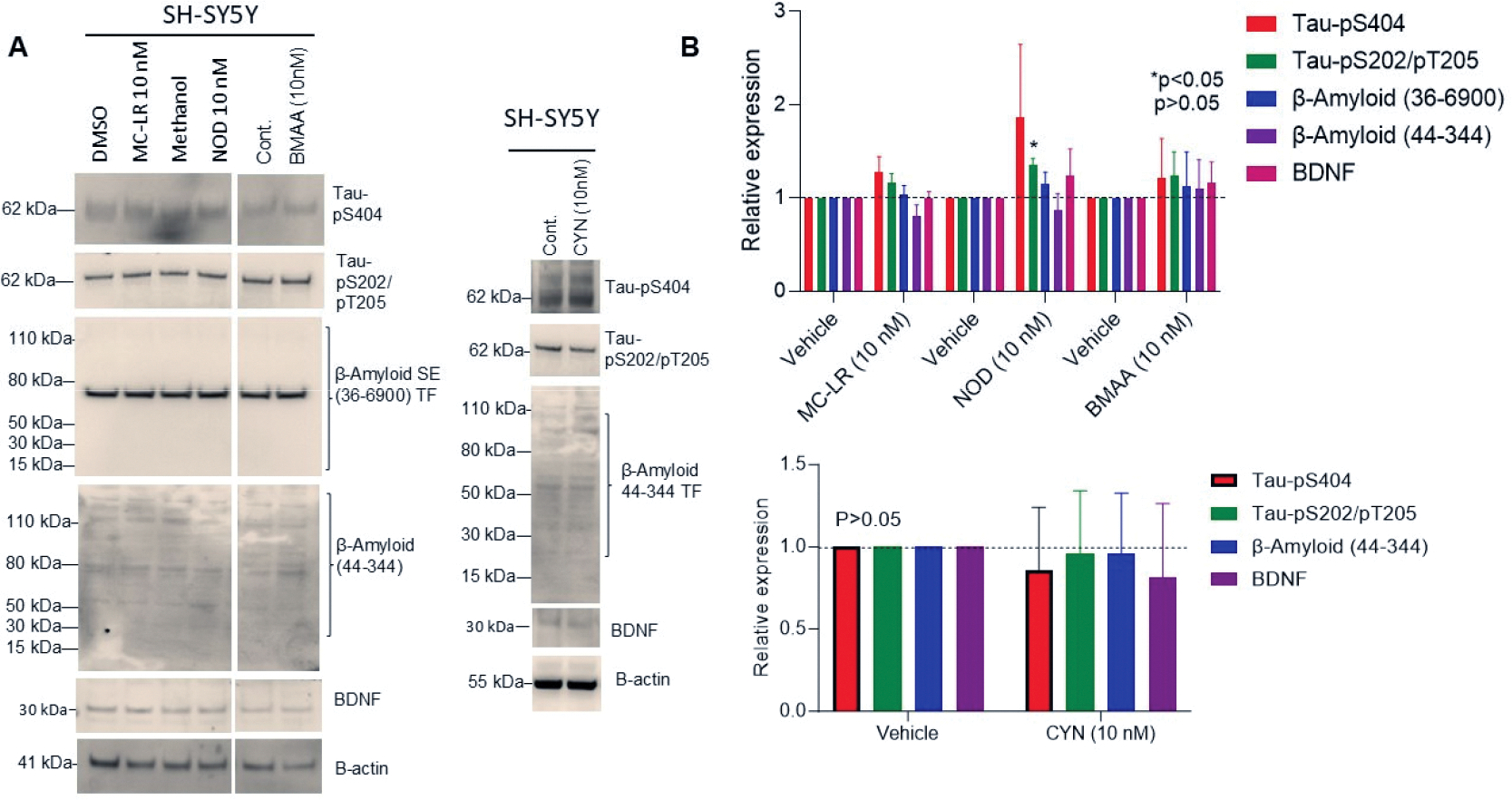
Effect of CT on AD biomarker analysis in SH-SY5Y cells. (**A&B**) SH-SY5Y cells were exposed to MC-LR, NOD, CYN, and BMAA at 10 nM concentration for 72 h. Sixty micrograms of protein lysates were immunoblotted with anti-Tau-pS404, anti-Tau-pS202-pT205, anti-β amyloid, anti-BDNF, and anti-actin antibodies. The band intensities were quantified by Image J and plotted (accessed on 25 June 2024). *P<0.05 compared with vehicle-exposed cells. p>0.05; ns: Not Significant.

To determine the association of CT exposure (MC-LR, NOD, CYN, and BMAA) with AD pathologic markers, we examined Tau phosphorylation levels, β-amyloid and BDNF protein expression (Figures 5A and 5B). Immunoblotting and band quantification revealed that MC-LR increased Tau-pS404, Tau-pS202/pT205 (ns), while NOD increased Tau-pS404 (p<0.05: ns), Tau-pS202/pT205 (p>0.05), β-amyloid (36–6900), and BDNF proteins (ns). BMAA exposure similarly increased Tau-pS404, Tau-pS202/pT205, β-amyloid, and BDNF protein expression (ns-showing higher mean values), although not significantly, compared to vehicle-treated cells (Figures 5A and 5B). In contrast, CYN exposure did not show any changes in Tau-pS404, Tau-pS202/pT205, β-amyloid, and BDNF protein expression as indicated compared with vehicle-treated cells (Figures 5A and 5B).

## Discussion

CTs are a group of harmful substances produced by aquatic blue-green algae, with more than 200 variants of microcystins (MCs) identified to date [37]. A major incident occurred in 1996, 52 people died and 130 people were subjected to renal hemodialysis treatment following accidental exposure to hepatotoxic CTs through contaminated water [38]. These toxins can accumulate in the human body through direct consumption of CT-contaminated drinking water, seafood, and associated food chains [37]. Inhalation exposure to CTs from terrestrial cyanobacteria also occurs, but less common [39]. Apart from hepatotoxicity, CTs can pass BBB and cause several detrimental changes in brain functions, Including altered calcium and protein homeostasis, neurotransmission, and behavioral changes [11,26]. Although the neurotoxic potential of certain CTs has been linked to the pathogenesis of neurodegenerative diseases, including Alzheimer’s disease (AD), the evidence connecting CTs directly to brain cancers remains limited, especially at low concentrations [28].

Neuroblastoma originates from the immature nervous system and is one of the most prevalent extracranial cancers (solid tumors) in children. Approximately 70% of patients experience metastasis within a year of diagnosis [40]. Several environmental pollutants, including air and water contaminants such as CTs, have been linked to an increased risk of neuroblastoma development in children [26,41]. Although CTs are well recognized for their hepatotoxic effects, compounds such as MCs, BMAA, guan toxin, anatoxin-a, and saxitoxin have also been shown to exert neurotoxicity [26]. Previous studies have demonstrated that chronic exposure to CTs such as MCs, NOD, and CYN can contribute to liver diseases and cancer [42–44], while exposure to MCs and BMAA has been associated with neurodegenerative diseases and neurotoxicity [32,45].

In this study, we investigated the cytotoxic effects of MC-LR, NOD, CYN, and BMAA at low concentrations (10 and 50 nM) on human neuroblastoma SH-SY5Y cells. Our findings show that acute exposure to these low doses increased cell survival and modulated metabolic activity. Interestingly, an earlier *in vitro* study indicated that concentrations exceeding 100 nM of CYN, MC-LR, and anatoxin-a (ATX-a), both individually and in combination, induced apoptosis in murine macrophage-like RAW264.7 cells, microglial BV-2 cells, and neuroblastoma N2a cells, suggesting a potential link to neurodegeneration [46]. In contrast, our findings indicate increased cell survival, possibly due to the lower concentrations of cyanotoxins used in this study, pointing to a dose-dependent effect of CTs. Moreover, our data demonstrate that these CTs affect/regulate inflammatory signaling, unfolded protein response (UPR), and mitochondrial energetics/activities even at low concentrations. Acute exposure to CTs (10 nM) not only upregulates cell survival but also regulates cell metabolic *CYP1A1*, *CYP1A2*, *CYP2D6*, *CYP2E1*, *CYP3A4*, and *VEGFA* gene expression in SH-SY5Y cells. Moreover, CTs affect the mitochondrial oxygen consumption rate, increase inflammatory *IL-6*, *SOD1*, and *TNFα* expression, and the UPR associated gene expression.

Numerous studies have shown that CTs at higher concentrations, compared to those used in our study, impact mitochondrial activity and induce cytotoxicity in neuronal cells. For instance, Hinojosa *et al.* demonstrated that exposure to cyanobacterial *Chrysosporum ovalisporum* (CYN+) extracts (CYN concentration at 0–1.11 μg/mL) showed a concentration and time-dependent (24–48h) reduction of cell viability (five-fold) in human neuroblastoma SH-SY5Y cells, compared with exposure to *Raphidiopsis raciborskii* (CYN) extract. Increased reactive oxygen species (ROS) production and reduced glutathione (GSH) levels were observed within 24 h following exposure to CYN, indicating that higher concentrations of CYN induce oxidative stress in SH-SY5Y cells [47]. Similarly, MC-LR has been shown to cause mitochondrial dysfunction in hippocampal neurons, leading to the accumulation of ROS. This mitochondrial dysfunction triggered the release of pro-inflammatory cytokines, ultimately resulting in neuronal apoptosis [48]. In mice hippocampus, MC-LR exposure (1, 5, 10 μg/L in drinking water) reduced mitochondrial DNA (mt-DNA) copy number, whereas at 20 and 40 μg/L concentration, an increase in mt-DNA copy number was observed in the cerebral cortex, likely reflecting a compensatory response to heightened mitochondrial oxidative stress [49]. Moreover, MC-LR at 0.1 μM was shown to alter hippocampal neuronal morphology and promote astrocyte proliferation and differentiation via the Hippo signaling pathway [49,50], suggesting that MC-LR exerts concentration-dependent effects on neuronal and glial cell populations.

We further assessed neuronal cytotoxicity by analyzing Tau phosphorylation, β-amyloid accumulation, and BDNF expression following exposure to low concentrations of CTs. Our results showed an increased Tau phosphorylation, β-amyloid, and BDNF protein expression (although not significantly), suggesting that these CTs modulate neurotoxicity and potentially contribute to neurodegenerative diseases. Our findings align with previous reports linking BMAA to progressive neurological disorders such as ALS, AD, and PD [28,31,32]. Similarly, CT MC-LR exposure has been shown to alter hippocampus protein expression involved in oxidative stress, apoptosis, cellular energy regulation, cytoskeletal development, and neurodegenerative disease pathways regulation [33]. These changes induced by MC-LR may lead to tau hyperphosphorylation, spatial memory impairment, and other neuronal degenerative changes, suggesting a potential role in the development of Alzheimer’s disease [33]. While our study employed an acute exposure model, additional research is needed to elucidate the long-term effects of chronic low-dose CT exposure, whether individual or combined, on the progression of neurodegenerative diseases.

While the current study suggests that lower concentrations of CTs affect inflammatory, UPR, mitochondrial respiration, and AD signaling, the effect is not significant. Acute exposure of CTs such as MC-LR and NOD regulated cell survival and metabolic activities in SH-SY5Y and N18TG2 cells; however, differential inflammatory and UPR gene responses were observed when cells were exposed to individual CTs. On the other hand, NOD and BMAA suppress OCR, suggesting that CTs induce cytotoxicity at a lower level in neuroblastoma cells. To determine the significant impact of lower concentrations of MC-LR, NOD, CYN, and BAMM separately or in combination, a chronic exposure approach will be a better strategy to assess the cytotoxicity exerted by different CTs.

## Conclusions

Although the concentrations of human exposure to CT vary, our data suggest that even at low concentrations of acute exposure to MC-LR, NOD, CYN, and BMAA change cell metabolic activities, increase inflammatory *IL-6*, *SOD1*, and *TNFα* gene expression, and cytotoxicity in neuroblastoma cells. CTs also affect mitochondrial oxygen consumption rate, UPR-associated gene expression, and increased Tau phosphorylation, β-amyloid, and BDNF protein expression. These results suggest that CTs may modulate neurotoxicity and contribute to Alzheimer’s disease (AD) pathogenesis, even at low concentrations. Our future studies will be investigating the effects of chronic CT exposure and their potential role in neurotoxicity and AD progression using animal models.

## Supplementary Material

Table S1

## Data Availability

The data set used and/or analyzed during the study is available to the corresponding authors upon request.

## Abbreviations:

CTs: Cyanotoxins
MC-LR: Microcystin-LR
NOD: Nodularin
CYN: Cylindrospermopsin
BMAA: β-N-methylamino-l-alanine
FBS: Fetal Bovine Serum
IL: Interleukin
MTT: 3-(4,5-Dimethylthiazol-2-yl)-2,5-diphenyltetrazolium bromide
PVDF: Polyvinylidene Difluoride
ROS: Reactive Oxygen Species
TNF-α: Tumor Necrosis Factor-α
OCR: Oxygen Consumption Rate

## References

[1] Bouaïcha N, Miles CO, Beach DG, Labidi Z, Djabri A, Benayache NY, Structural Diversity, Characterization and Toxicology of Microcystins. Toxins. 2019 Dec 7;11(12):714. 31817927 10.3390/toxins11120714 PMC6950048

[2] Chorus I, Welker M. Toxic cyanobacteria in water: a guide to their public health consequences, monitoring and management. Taylor & Francis; 2021.

[3] Flores NM, Miller TR, Stockwell JD. A Global Analysis of the Relationship between Concentrations of Microcystins in Water and Fish. Frontiers in Marine Science. 2018 Feb 9;5:30.

[4] Greer B, Meneely JP, Elliott CT. Uptake and accumulation of Microcystin-LR based on exposure through drinking water: An animal model assessing the human health risk. Sci Rep. 2018 Mar 20;8(1):4913. 29559706 10.1038/s41598-018-23312-7 PMC5861052

[5] Luukkainen R, Namikoshi M, Sivonen K, Rinehart KL, Niemelä SI. Isolation and identification of 12 microcystins from four strains and two bloom samples of Microcystis spp.: structure of a new hepatotoxin. Toxicon. 1994 Jan;32(1):133–9. 9237346 10.1016/0041-0101(94)90030-2

[6] Farrer D, Counter M, Hillwig R, Cude C. Health-based cyanotoxin guideline values allow for cyanotoxin-based monitoring and efficient public health response to cyanobacterial blooms. Toxins. 2015 Feb 5;7(2):457–77. 25664510 10.3390/toxins7020457 PMC4344635

[7] Kenefick SL, Hrudey SE, Peterson HG, Prepas EE. Toxin release from Microcystis aeruginosa after chemical treatment. Water Science and Technology. 1993 Feb 1;27(3–4):433–40.

[8] Gu S, Jiang M, Zhang B. Microcystin-LR in Primary Liver Cancers: An Overview. Toxins. 2022 Oct 20;14(10):715. 36287983 10.3390/toxins14100715 PMC9611980

[9] Abdallah MF, Van Hassel WHR, Andjelkovic M, Wilmotte A, Rajkovic A. Cyanotoxins and Food Contamination in Developing Countries: Review of Their Types, Toxicity, Analysis, Occurrence and Mitigation Strategies. Toxins (Basel). 2021 Nov 6;13(11):786. 34822570 10.3390/toxins13110786 PMC8619289

[10] Lad A, Breidenbach JD, Su RC, Murray J, Kuang R, Mascarenhas A, As We Drink and Breathe: Adverse Health Effects of Microcystins and Other Harmful Algal Bloom Toxins in the Liver, Gut, Lungs and Beyond. Life (Basel). 2022 Mar 14;12(3):418. 35330169 10.3390/life12030418 PMC8950847

[11] Chatterjee S, More M. Cyanobacterial Harmful Algal Bloom Toxin Microcystin and Increased Vibrio Occurrence as ClimateChange-Induced Biological Co-Stressors: Exposure and Disease Outcomes via Their Interaction with Gut-Liver-Brain Axis. Toxins (Basel). 2023 Apr 17;15(4):289. 37104227 10.3390/toxins15040289 PMC10144574

[12] Drobac D, Tokodi N, Simeunović J, Baltić V, Stanić D, Svirčev Z. Human exposure to cyanotoxins and their effects on health. Arh Hig Rada Toksikol. 2013 Jun;64(2):119–30. 23819940 10.2478/10004-1254-64-2013-2320

[13] Svirčev Z, Chen L, Sántha K, Drobac Backović D, Šušak S, Vulin A, A review and assessment of cyanobacterial toxins as cardiovascular health hazards. Arch Toxicol. 2022 Nov;96(11):2829–63. 35997789 10.1007/s00204-022-03354-7 PMC9395816

[14] Niture S, Gadi S, Qi Q, Rios-Colon L, Khatiwada S, Vandana, Cyanotoxins Increase Cytotoxicity and Promote Nonalcoholic Fatty Liver Disease Progression by Enhancing Cell Steatosis. Toxins (Basel). 2023 Jun 25;15(7):411. 37505679 10.3390/toxins15070411 PMC10467139

[15] Hernandez BY, Zhu X, Nagata M, Loo L, Chan O, Wong LL. Cyanotoxin exposure and hepatocellular carcinoma. Toxicology. 2023 Mar 15;487:153470. 36863303 10.1016/j.tox.2023.153470 PMC10358828

[16] Gutiérrez-Praena D, Guzmán-Guillén R, Pichardo S, Moreno FJ, Vasconcelos V, Jos Á, Cytotoxic and morphological effects of microcystin-LR, cylindrospermopsin, and their combinations on the human hepatic cell line HepG2. Environ Toxicol. 2019 Mar;34(3):240–51. 30461177 10.1002/tox.22679

[17] Kumar P, Hegde K, Brar SK, Cledon M, Kermanshahi-Pour A. Potential of biological approaches for cyanotoxin removal from drinking water: A review. Ecotoxicol Environ Saf. 2019 May 15;172:488–503. 30738231 10.1016/j.ecoenv.2019.01.066

[18] Schneider M, Bláha L. Advanced oxidation processes for the removal of cyanobacterial toxins from drinking water. Environmental Sciences Europe. 2020 Dec;32(1):94.

[19] Chen H, Burke JM, Prepas EE. Cyanobacterial Toxins in Fresh Waters. In: Nriagu JO, Editor. Encyclopedia of Environmental Health. Burlington; Elsevier; 2011. p. 860–71.

[20] Lauritano C, Carotenuto Y, Roncalli V. Glutathione S-transferases in marine copepods. Journal of Marine Science and Engineering. 2021 Sep;9(9):1025.

[21] Zhou J, Wang WN, Wang AL, He WY, Zhou QT, Liu Y, Glutathione S-transferase in the white shrimp Litopenaeus vannamei: Characterization and regulation under pH stress. Comp Biochem Physiol C Toxicol Pharmacol. 2009 Aug;150(2):224–30. 19426830 10.1016/j.cbpc.2009.04.012

[22] Galanti LN, Amé MV, Wunderlin DA. Accumulation and detoxification dynamic of cyanotoxins in the freshwater shrimp Palaemonetes argentinus. Harmful Algae. 2013 Jul 1;27:88–97.

[23] Cai D, Wei J, Huang F, Feng H, Peng T, Luo J, The detoxification activities and mechanisms of microcystinase towards MC-LR. Ecotoxicol Environ Saf. 2022 May 1;236:113436. 35367885 10.1016/j.ecoenv.2022.113436

[24] Wang J, Zhang C, Zhu J, Ding J, Chen Y, Han X. Blood-brain barrier disruption and inflammation reaction in mice after chronic exposure to Microcystin-LR. Sci Total Environ. 2019 Nov 1;689:662–78. 31279213 10.1016/j.scitotenv.2019.06.387

[25] Feurstein D, Holst K, Fischer A, Dietrich DR. Oatp-associated uptake and toxicity of microcystins in primary murine whole brain cells. Toxicol Appl Pharmacol. 2009 Jan 15;234(2):247–55. 19027771 10.1016/j.taap.2008.10.011

[26] Metcalf JS, Tischbein M, Cox PA, Stommel EW. Cyanotoxins and the Nervous System. Toxins (Basel). 2021 Sep 16;13(9):660. 34564664 10.3390/toxins13090660 PMC8472772

[27] Hedrick E, Tiwari A, Niture S, Cheng Q, Kumar D, Mukhopadhyay S. Microcystin: From Blooms to Brain Toxicity. J Cell Signal. 2025;6(1):29–38. 40534901 10.33696/Signaling.6.131 PMC12176425

[28] Tischbein M, Stommel EW. Neurotoxic Cyanobacterial Toxins. In: Kostrzewa RM, Editor. Handbook of Neurotoxicity. Cham: Springer International Publishing; 2022 Jan 11. pp. 1–28.

[29] Lopicic S, Svirčev Z, Palanački Malešević T, Kopitović A, Ivanovska A, Meriluoto J. Environmental Neurotoxin β-N-MethylaminoL-alanine (BMAA) as a Widely Occurring Putative Pathogenic Factor in Neurodegenerative Diseases. Microorganisms. 2022 Dec 6;10(12):2418. 36557671 10.3390/microorganisms10122418 PMC9781992

[30] Hinojosa MG, Gutiérrez-Praena D, Prieto AI, Guzmán-Guillén R, Jos A, Cameán AM. Neurotoxicity induced by microcystins and cylindrospermopsin: A review. Sci Total Environ. 2019 Jun 10;668:547–65. 30856566 10.1016/j.scitotenv.2019.02.426

[31] Chiu AS, Gehringer MM, Welch JH, Neilan BA. Does α-aminoβ-methylaminopropionic acid (BMAA) play a role in neurodegeneration? Int J Environ Res Public Health. 2011 Sep;8(9):3728–46. 22016712 10.3390/ijerph8093728 PMC3194113

[32] Pablo J, Banack SA, Cox PA, Johnson TE, Papapetropoulos S, Bradley WG, Cyanobacterial neurotoxin BMAA in ALS and Alzheimer’s disease. Acta Neurol Scand. 2009 Oct;120(4):216–25. 19254284 10.1111/j.1600-0404.2008.01150.x

[33] Li G, Cai F, Yan W, Li C, Wang J. A proteomic analysis of MCLR-induced neurotoxicity: implications for Alzheimer’s disease. Toxicol Sci. 2012 Jun;127(2):485–95. 22430071 10.1093/toxsci/kfs114

[34] Sidell N Retinoic acid-induced growth inhibition and morphologic differentiation of human neuroblastoma cells in vitro. J Natl Cancer Inst. 1982 Apr;68(4):589–96. 7040765

[35] Pulido OM, Rousseaux CG, Cole PI. Food and toxicologic pathology. In: Haschek WM, Rousseaux CG, Wallig MA, Bolon B, Editors. Haschek and Rousseaux’s Handbook of Toxicologic Pathology. 4th Edn. New York: Academic Press; 2023 Jan 1. pp 33–103.

[36] Scheper W, Hoozemans JJ. The unfolded protein response in neurodegenerative diseases: a neuropathological perspective. Acta Neuropathol. 2015 Sep;130(3):315–31. 26210990 10.1007/s00401-015-1462-8 PMC4541706

[37] Al Haffar M, Fajloun Z, Azar S, Sabatier JM, Abi Khattar Z. Lesser-Known Cyanotoxins: A Comprehensive Review of Their Health and Environmental Impacts. Toxins (Basel). 2024 Dec 19;16(12):551. 39728809 10.3390/toxins16120551 PMC11680425

[38] Azevedo SM, Carmichael WW, Jochimsen EM, Rinehart KL, Lau S, Shaw GR, Human intoxication by microcystins during renal dialysis treatment in Caruaru-Brazil. Toxicology. 2002 Dec 27;181–182:441–6. 10.1016/s0300-483x(02)00491-2 12505349

[39] Metcalf JS, Richer R, Cox PA, Codd GA. Cyanotoxins in desert environments may present a risk to human health. Sci Total Environ. 2012 Apr 1;421–422:118–23. 10.1016/j.scitotenv.2012.01.053 22369867

[40] Salemi F, Alam W, Hassani MS, Hashemi SZ, Jafari AA, Mirmoeeni SMS, Neuroblastoma: Essential genetic pathways and current therapeutic options. Eur J Pharmacol. 2022 Jul 5;926:175030. 35605657 10.1016/j.ejphar.2022.175030

[41] Heck JE, Park AS, Qiu J, Cockburn M, Ritz B. An exploratory study of ambient air toxics exposure in pregnancy and the risk of neuroblastoma in offspring. Environ Res. 2013 Nov;127:1–6. 24139061 10.1016/j.envres.2013.09.002 PMC3960946

[42] Nishiwaki-Matsushima R, Ohta T, Nishiwaki S, Suganuma M, Kohyama K, Ishikawa T, Liver tumor promotion by the cyanobacterial cyclic peptide toxin microcystin-LR. J Cancer Res Clin Oncol. 1992;118(6):420–4. 1618889 10.1007/BF01629424 PMC12200900

[43] Falconer IR, Humpage AR. Preliminary evidence for in vivo tumour initiation by oral administration of extracts of the blue-green alga cylindrospermopsis raciborskii containing the toxin cylindrospermopsin. Environ Toxicol. 2001;16(2):192–5. 11339720 10.1002/tox.1024

[44] Vankova DG, Pasheva MG, Kiselova-Kaneva YD, Ivanov DL, Ivanova DG. Mechanisms of Cyanotoxin Toxicity—Carcinogenicity, Anticancer Potential, and Clinical Toxicology. In: Pınar E, Tomohisa O, Editors. Medical Toxicology.. Rijeka: IntechOpen; 2019 Jul 16. p. Ch. 8.

[45] Hu Y, Chen J, Fan H, Xie P, He J. A review of neurotoxicity of microcystins. Environ Sci Pollut Res Int. 2016 Apr;23(8):7211–9. 26857003 10.1007/s11356-016-6073-y

[46] Takser L, Benachour N, Husk B, Cabana H, Gris D. Cyanotoxins at low doses induce apoptosis and inflammatory effects in murine brain cells: Potential implications for neurodegenerative diseases. Toxicology reports. 2016 Jan 1;3:180–9. 28959538 10.1016/j.toxrep.2015.12.008 PMC5615428

[47] Hinojosa MG, Cascajosa-Lira A, Prieto AI, Gutiérrez-Praena D, Vasconcelos V, Jos A, Cytotoxic Effects and Oxidative Stress Produced by a Cyanobacterial Cylindrospermopsin Producer Extract versus a Cylindrospermopsin Non-Producing Extract on the Neuroblastoma SH-SY5Y Cell Line. Toxins (Basel). 2023 May 5;15(5):320. 37235355 10.3390/toxins15050320 PMC10223116

[48] Zhang C, Wang J, Zhu J, Chen Y, Han X. Microcystin-leucine-arginine induced neurotoxicity by initiating mitochondrial fission in hippocampal neurons. Sci Total Environ. 2020 Feb 10;703:134702. 31753492 10.1016/j.scitotenv.2019.134702

[49] Wang X, Xu L, Li X, Chen J, Zhou W, Sun J, The differential effects of microcystin-LR on mitochondrial DNA in the hippocampus and cerebral cortex. Environ Pollut. 2018 Sep;240:68–76. 29729571 10.1016/j.envpol.2018.04.103

[50] Xue Q, Yan Y, Zhang K, Zhang H, Zhao Y. Exposure to microcystin-LR promotes astrocyte proliferation both in vitro and in vivo via Hippo signaling pathway. Ecotoxicol Environ Saf. 2024 Jul 1;279:116480. 38772146 10.1016/j.ecoenv.2024.116480

