# Supplementary material for: Cytotoxicity Modulated by Cyanotoxins in Neuroblastoma SH-SY5Y Cells
Source: J Exp Pathol (Wilmington). Author manuscript; Available in PMC 2025 Dec 21. (PMC12717869; doi:10.33696/pathology.6.058)
Supplement: Table S1 [file NIHMS2123549-supplement-Table_S1.pdf]

Niture S, Gadi S, Mukhopadhyay S, Kumar D, Cheng Q. Cytotoxicity Modulated by Cyanotoxins in Neuroblastoma SH-SY5Y Cells. J Exp Pathol. 2025;6(1):29–39.

Supplementary Table 1

Set of primers used in the current study in RT/qPCR.

Primer sequence
GAPDH FP: 5′-CCACCCAGAAGACTGTGGAT -3′<br>GAPDH RP: 5′-GTTGAAGTCAGAGGAGACCACC-3′
CYP1A1 FP: 5′-GGTCTTTCTCTTCCTGGCTATC -3′<br>CYP1A1 RP: 5′-CTGTCTCTTCCCTTCACTCTTG -3′
CYP1A2 FP: 5′-CAGGAGCACTATCAGGACTTTG -3′<br>CYP1A2 RP: 5′-GTTGACCTGCCACTGGTTTA -3′
CYP2E1 FP: 5′-CTGAGAAGTTCCTCCCTGAAAG -3′<br>CYP2E1 RP: 5′-AGAAGCAGAGGAGCCAAATC -3′
CYP2D6 FP: 5′-CTAAGGGAACGACACTCATCAC -3′<br>CYP2D6 RP: 5′-AGGGAGGTGAAGAAGAGGAA -3′
CYP3A4 FP: 5′-CTGAGAAGTTCCTCCCTGAAAG -3′<br>CYP3A4 RP: 5′-AGAAGCAGAGGAGCCAAATC -3′
VEGFA FP: 5′-TGGTGTCTTCACTGGATGTATTT -3′<br>VEGFA RP: 5′-CCTGTACCTGTGATCTGTCTTTC -3′
SOD1 FP: 5′-GTGTGGCCGATGTGTCTATT -3′<br>SOD1 RP: 5′-CTCAGACTACATCCAAGGGAATG -3′
NAT1(3) FP: 5′-GATGTGAACTGCAACTCCAAAG -3′<br>NAT1(3) RP: 5′-GCCCAGTACAGAAGATGATTGA -3′
IL6 FP: 5′-GTAGTGAGGAACAAGCCAGAG-3′<br>IL6 RP: 5′-GGACTGCAGGAACTCCTTAAA-3′
IL8 FP: 5′-AGACAGCAGAGCACACAAG<br>IL8 RP: 5′-GGGTGGAAAGGTTTGGAGTAT
TNFα FP: 5′- GAGCCAGCTCCCTCTATTTATG-3′<br>TNFα RP: 5′- AGGGCGATTACAGACACAAC-3′
HMOX1 FP: 5′-GGTCCTTACACTCAGCTTTCT -3′<br>HMOX1 RP: 5′-CATAGGCTCCTTCCTCCTTTC -3′
CAT FP: 5′-CCTCTCATCCCAGTTGGTAAAC<br>CAT RP: 5′-TGTTGAATCTCCGCACTTCTC
eIF2a FP: 5′-CCTTCTGCTGCCTCTAAGATAAA-3′<br>eIF2a RP: 5′-GGGAGCTGAGTGCTACAATAAA-3′
IRE1a FP: 5′-GTGGCCTTCATCATCACCTATC-3′<br>IRE1a RP: 5′-CCTCATCTCCATCGTCTTGTTC-3′
ATF4 FP: 5′-CCATGATCCCTCAGTGCATAA-3′<br>ATF4 RP: 5′-TGCGGACCTCTTCTATCAAATC-3′
BIP FP: 5′-CCTTCGATGTGCCTCTTCTCAC-3′

BIP RP: 5′-GGACGGGCTTCATAGTA-3′
ATF6 FP: 5′-GAACTTCGAGGATGGGTTCATAG-3′<br>ATF6 RP: 5′-GTGGTCTTGTTATGGGTGGTGGTAG-3′
